# Copper conductive patterns through spray-pyrolysis of copper-diethanolamine complex solution

**DOI:** 10.1371/journal.pone.0200084

**Published:** 2018-07-03

**Authors:** Jutamart Chotipanich, Suraya Hanim Abu Bakar, Amornchai Arponwichanop, Tetsu Yonezawa, Soorathep Kheawhom

**Affiliations:** 1 Department of Chemical Engineering, Faculty of Engineering, Chulalongkorn University, Bangkok, Thailand; 2 Computational Process Engineering Research Unit, Chulalongkorn University, Bangkok, Thailand; 3 Division of Materials Science and Engineering, Faculty of Engineering, Hokkaido University, Sapporo, Hokkaido, Japan; Istituto Italiano di Tecnologia, ITALY

## Abstract

A simple and low-cost method to fabricate copper conductive patterns at low temperature is critical for printed electronics. Low-temperature spray-pyrolysis of copper-alkanolamine complex solution shows high potential for this application. However, the produced copper patterns exhibit a granular structure consisting of connected fine copper particles. In this work, low-temperature spray-pyrolysis of copper formate-diethanolamine complex solution under N_2_ flow at a temperature of 200 °C was investigated. The effects of spraying conditions on microstructure and electrical properties of the patterns were examined. Our results revealed that the spraying rate is a critical parameter determining the degree of sintering and electrical resistivity of the patterns. A low spraying rate facilitates sintering, and hence well-sintered copper patterns with the lowest resistivity of 6.12 *μ*Ω.cm (3.6 times of bulk copper) on a polyimide substrate could be fabricated.

## Introduction

Photolithography combining with wet processing (Developing Etching Stripping, DES) has been used commonly for the manufacture of a conductive pattern which is an essential element in all electronic devices. However, this method is time-consuming and expensive. Besides, it produces a significant amount of chemical wastes.

Printing technology which is an additive manufacturing technique has been extensively investigated as a promising method for the fabrication of electronic devices. This technique is simple, inexpensive, high throughput and environmental friendly [1–4]. Commonly used printing techniques include ink-jet printing and screen printing [5]. Thermal spray coating is one of the most simple and cost effective methods to deposit materials in thin film form [6, 7]. Though this technique is not inherently patternable as other printing techniques, the patterns can be efficiently fabricated by shadow masking. This technique has been widely applied to deposit various metal oxides and inorganic compounds such as copper oxide [8] and copper iodide [9]. Also, fabrication of copper thin films were reported [10, 11]. Nevertheless, the films fabricated through this technique often show a non-uniform granular and porous structure consisting of connected fine particles [12]. To address this issue, enormous research effort has been devoted [13, 14].

Copper (Cu) is considered the best material for fabrication of electrically conductive patterns because of its low cost, high electrical conductivity, and excellent property of electro-migration. However, its tendency to form a nonconductive oxide film hinders its practical application in printed electronics [15–19].

Recently, Cu complex inks, also known as copper-based metal-organic decomposition (MOD) inks, have been extensively investigated [20]. The complex inks are more resistant to oxidation in air. Moreover, the problems such as nozzle clogging can be overcome. After printing, the inks require post-processing to convert copper-ion precursors to metallic copper and simultaneously form a connected conductive pathway. Recent works reported that the inks could be processed at low temperature [21–32].

Lee et al. [25] studied microstructure and electrical property of the copper films fabricated by thermal and laser sintering of a copper complex ink made of copper formate, isopropyl alcohol, hexylamine, and 2-amino-2-methyl-1-propanol. The results showed that the laser-sintered film exhibited a much tighter structure whilst the thermal-sintered film had a better crystalline quality. However, the minimum resistivity values obtained from both methods were almost identical.

Kim et al. [22] investigated fabrication of copper films from a complex ink of copper formate and hexylamine. The films were annealed at 200 °C under an inert atmosphere and followed by reduction with formic acid gas at 250 °C. It was reported that copper concentration in the ink significantly affected the impurity content and the microstructure of the films.

Cho et al. [31] studied a self-reducible copper complex ink composed by copper formate, alkanolamines and polyalcohols. They reported that glycerol was the most suitable reduction assistant-material because of its relatively abundant hydroxyl groups, excellent evaporation properties, and environmentally friendly. The copper film with a resistivity of 17 *μ*Ω.cm was fabricated by thermal sintering at 350 °C in ambient air.

One major problem preventing widespread application of copper complex inks is the notorious coffee ring effect. Once the printed patterns are heated, a vapor recoiling force, induced by solvent evaporation at the interface, tends to push copper particles, generated from decomposition of the inks, into isolated clusters. By using spray-pyrolysis, evaporation of solvents as well as decomposition of the complex inks occurred inside fine aerosol droplets. Thus, the coffee ring effect can be entirely avoided.

Previously, fabrication of thin copper films using spray-pyrolysis was reported [11]. Bis-acetyalacetonato copper, bis-hexafluroacetylacetonato copper and bis-dipivaloylmethanato copper were used as copper precursors. The copper films were fabricated at 250-400 °C for 3-12 h under a mixture of ultrahigh pure N_2_ and H_2_. However, this technique is not suitable for printed electronics as the polymer substrates cannot tolerate such a high processing temperature for a long time. Besides, spray-pyrolysis also applied in the fabrication of copper oxide thin films using cupric acetate as a copper precursor [8]. Though the resulted films were not electrically conductive, optical properties of the films and the effects of substrate and annealing temperature were investigated.

Later, fabrication of copper-silver films using spray-pyrolysis at low temperature under a N_2_ atmosphere was demonstrated [10]. The ink used was prepared from copper acetate, silver oxide, ammonia solution and diethanolamine. At spray-pyrolysis temperature 200 °C, the conductive film with a minimum resistivity of 19 *μ*Ω.cm could be fabricated. In spite of copper formate, which is considered more hazardous and corrosive, copper acetate was used. Nevertheless, as silver was introduced to reduce the resistivity of the conductive films, the added silver adversely affected electro-migration resistivity of the conductive films. Though copper acetate is more environmentally favorable, copper formate is more favorable regarding the resistivity of the resulted film and the processing temperature [30]. Besides, copper formate has low organic content. Thus inks with high copper load can be prepared.

In this study, we focus on investigating the fabrication of conductive copper patterns using spray-pyrolysis of copper formate-diethanolamine complex solution under N_2_ flow. The maximum temperature of 200 °C was considered. Reaction and thermal analysis of the solution were investigated. Besides, the effects of spray-pyrolysis temperature, annealing time and spraying rate on microstructure and electrical properties of the patterns were examined.

## Experimental

### Preparation of the copper complex ink

Copper (I) oxide (99.0%, Sigma Aldrich), formic acid (85.0%, QRëCTM) and ethanol (99.9%, QRëCTM) were used to synthesize copper (II) formate tetrahydrate, copper-ion precursor. Diethanolamine (HN(CH_2_CH_2_OH)_2_, DEA, 98.5% purity) and ammonia solution (NH_4_OH, 25% wt/wt, Ajax Finechem) were used to prepare the copper complex ink. These chemicals were used in a as-received form without further purification. Polyimide (PI, Dupont Kapton 100HN 25 *μ*m.) were used as substrates.

Copper formate was synthesized by precipitation from the reaction of copper oxide with formic acid. 20 g of copper oxide powder was put into 200 ml of formic acid and the mixture was vigorously stirring at room temperature in an ambient atmosphere for 1 h. As a result of the reaction, the color of the mixture changed from black to sky-blue. The synthesized copper formate was separated by filtering the mixture and washed with ethanol. The product was dried at 40 °C for 5 h.

**Fig 1:**
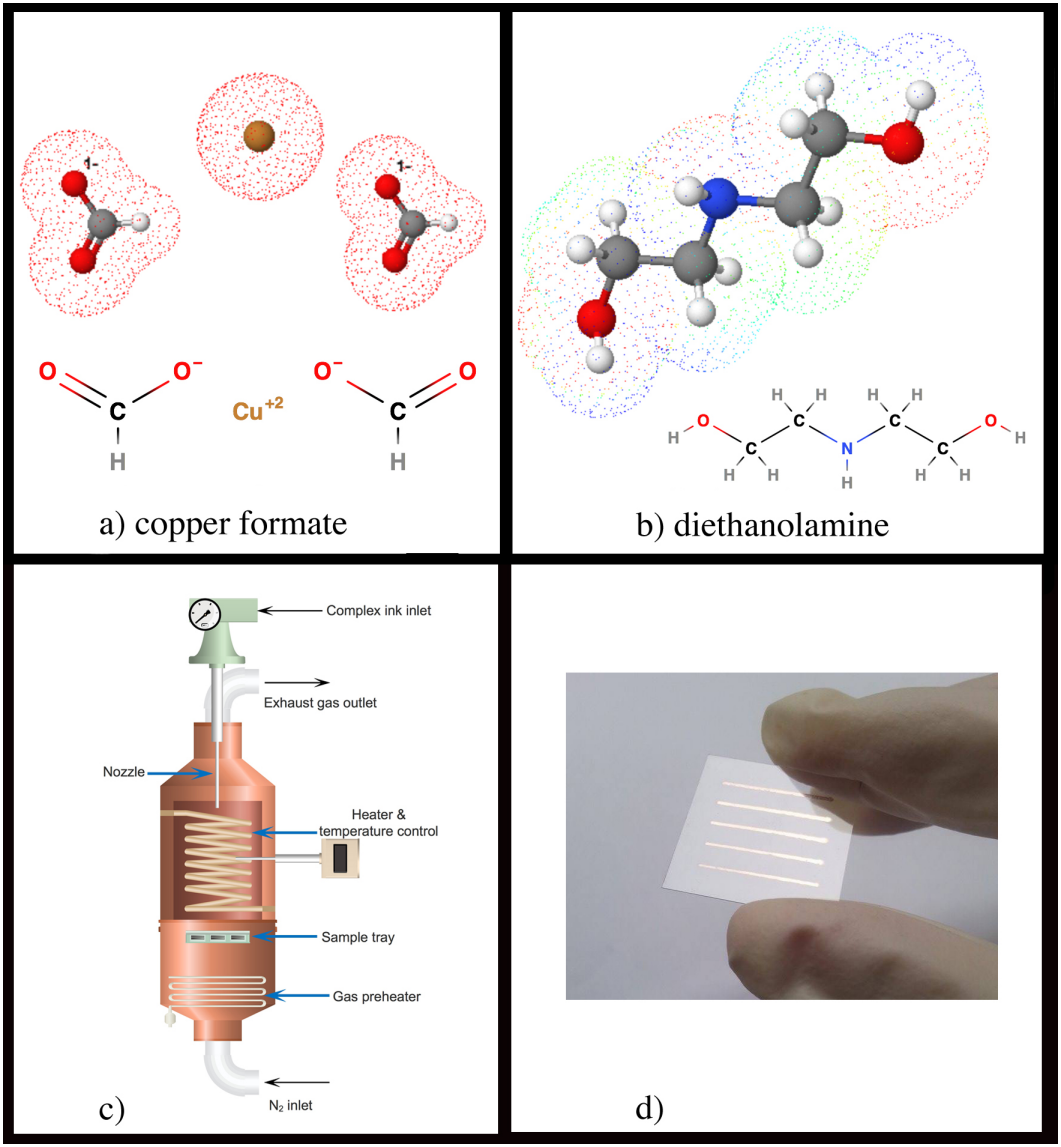
Molecular structures of copper formate and diethanolamine, spray-pyrolysis and a photographic image of the samples. (a) molecular structure of copper formate. (b) molecular structure of diethanolamine. (c) schematic setup of spray-pyrolysis process. (d) photographic image of the fabricated copper pattern.

The complex inks were prepared by mixing a varying amount of synthesized copper formate (0.338 g (1.5 mmol), 0.790 g (3.5 mmol), 1.128 g (5.0 mmol) and 1.580 g (7.0 mmol)) in 5 mL of 25% wt/wt ammonia solution. These solutions were then mixed with 1 mL of deionized (DI) water and 4 mL of DEA. The solution obtained are the copper complex inks containing 0.15, 0.35, 0.50 and 0.70 M copper formate. The chemical structures of copper formate and DEA are presented in Fig 1a and 1b. DEA, polydentate ligand, can form multiple bonds with copper ions. In this case, the inks contain excessive amount of DEA ligands.

### Fabrication of the copper patterns by spray-pyrolysis

The spray-pyrolysis system is mainly composed of an air blast atomizer, a reaction chamber, and heaters. The setup for spray-pyrolysis system used in this work is shown in Fig 1c. The system mainly consists of an air blast atomizer made of stainless steel, a stainless steel reaction chamber, heater and gas preheater. In the atomizer, high speed nitrogen gas is used to produce an aerosol. The reaction chamber is a vertical cylindrical tube of diameter 20 cm and of length 30 cm. The heater with temperature control is installed at the wall of the chamber. Also, the gas preheater is installed below the substrate holder made of stainless steel mesh of diameter 9 cm and of thickness 0.2 cm. The maximum temperature of 250 °C can be attained over a uniform cylindrical zone inside the chamber. The substrate holder is placed in the uniform zone of the chamber. The temperature of the furnace is maintained and controlled with a temperature sensitivity of 1 °C.

In each experiment, conductive patterns were fabricated on 2.5 cm. × 2.5 cm PI substrate by spray-pyrolysis of 10.0 mL synthesized complex ink at 150 or 200 °C under N_2_ flow. The patterns consisting of five parallel lines (1 mm × 20 mm), as shown in Fig 1d, were fabricated using a shadow mask. The mask was 50 *μ*m thick with a tapered edge. Initially, the chamber was flushed with N_2_ at a flow rate of 6.0 L/min for 15 min to remove the air from the chamber and reach an inert atmosphere. Subsequently, 6.0 L/min of N_2_ was continuously fed to the chamber and the complex inks were sprayed at a different spraying rate (3.33, 2, 1 and 0.67 mL/min) with correspondent spraying time (3, 5, 10 and 15 min). Also, the moving arrangements of an atomizer were employed during spraying in order to achieve uniform deposition. The samples were then further annealed inside the chamber for a different annealing time 25 to 40 min while 6.0 L/min of N_2_ was continuously being fed into to the chamber during the annealing process.

### Characterization and measurement

Thermogravimetric/Differential Thermal Analysis (TG/DTA; PerkinElmer Inc., Diamond TG/DTA) was used to evaluate thermal decomposition behavior of the complex ink. Each complex ink was dropped in a pan for thermal analysis which was then subjected to a flow rate of 20 mL/min of N_2_ gas at a temperature ranging from 30 °C to 300 °C at a heating rate of 10 °C/min.

The morphologies of surfaces and cross-sections of the conductive patterns were observed using a field emission scanning electron microscope (FESEM; JEOL, JSM-7610F). The crystalline structure of the patterns was analyzed by X-ray diffractometer (XRD; Bruker, D2 PHASER). Samples were measured at 2*θ* from 20 to 80°.

The electrical resistivity of the conductive patterns was measured using a line scratching technique [33]. A pair of gold electrodes was sputtered through a mask over each line array so that the lines spanned the distance of 10 mm between electrodes. Four-point-probe measurements (Keithley Instruments, 2182A digital nanovoltmeter) were carried out to determine the total resistance of the five lines. Each line was carefully scratched off. Then, the new resistivity value was determined. Under the assumption that each line array is a parallel resistor network, this method allowed measuring the average resistance of each cut line. Besides, the thickness of the patterns was determined by examination of its cross-sectional SEM image.

## Results and discussion

### Reaction and thermal analysis of the complex inks

In this work, copper formate was used as the copper-ion precursor. Copper formate can be thermally decomposed to yield metallic copper and simultaneously generate hydrogen (H_2_) gas, as shown in (1) [34]. This facilitates the reduction of Cu^2+^ from copper formate to Cu(0), and prevents the oxidation of copper during annealing process.

$$\begin{array}{c} \text{Cu}(\text{HCOO}{)}_{2}\to \text{Cu}(\text{s})+{\text{H}}_{2}(\text{g})+2{\text{CO}}_{2}(\text{g}) \end{array}$$ (1)

Also, like other alkanolamines [27], DEA acts as a weak base, and forms bidentate bond with copper. Copper complexes of DEA are advantageous owing to their high stability during processing and storage. Also, the decomposition of the complexes occurs at sufficiently low temperatures. Moreover, alkanolamines also show reduction abilities and facilitates the reduction of Cu^2+^ [35]. Besides, DEA can be decomposed, as shown in (2) [36]. Formaldehyde generated from decomposition of DEA can also react with copper ions to form copper particles [37].

$$\begin{array}{c} \text{HN}({\text{CH}}_{2}{\text{CH}}_{2}\text{OH}{)}_{2}+2{\text{OH}}^{-}+2{\text{H}}_{2}\text{O}\to 2\text{HCHO}+2{\text{HCOO}}^{-}+{\text{NH}}_{3} \end{array}$$ (2)

**Fig 2:**
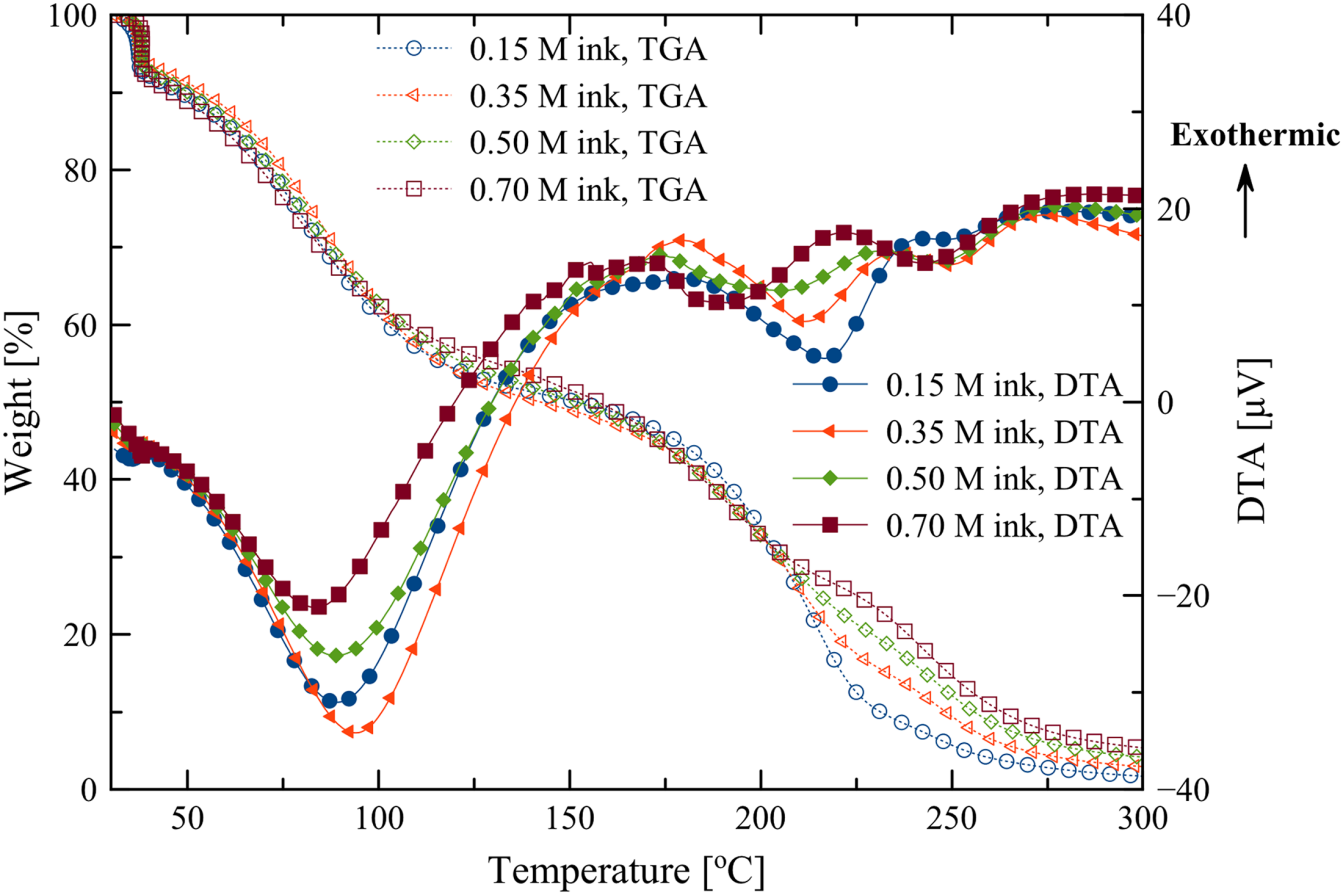
TG/DTA data of the inks with various concentrations of Cu-ion.

The pyrolysis temperature used in this work was determined by TG/DTA data as shown in Fig 2. In all cases, similar thermogravimetric patterns were observed. The first mass losses occurred below 50 °C are mainly attributed to evaporation of ammonia. The mass losses occurred between 50–125 °C are attributed to evaporation of water. Thermal decomposition of the copper complex and evaporation of excess DEA are occurred from 150 to 250 °C. The mass curves then reach a plateau at 275 °C, indicating total evaporation of excess DEA and the thermal decomposition of the copper complex. Then, the weights remained almost constant as the patterns densified at higher temperatures. DTA curves of all the inks also revealed two endothermic peaks. The small valley peaks around 40 °C mainly attributed to the evaporation of ammonia. The broad valley peaks around 90 °C mainly attributed to the evaporation of water whilst the relatively smaller valley peaks occurred at the temperature around 200 °C corresponded to the thermal decomposition of the copper complex and evaporation of excess DEA.

The results suggested that thermal decomposition of the copper complex occurred at temperatures greater than the evaporation temperature of the solvent. Thus, copper particles were generated in the absence of solvent. However, the evaporation of excess DEA occurred at the temperature close to decomposition temperature of the copper complex. Though the decomposition of the copper complex and evaporation of excess DEA take place between 150–250 °C, spray-pyrolysis temperatures between 140 and 200 °C were chosen because the substrate cannot tolerate temperature higher than 200 °C. After spraying, annealing at the same temperature of spraying was implemented.

### Effects of spray-pyrolysis temperature

**Fig 3:**
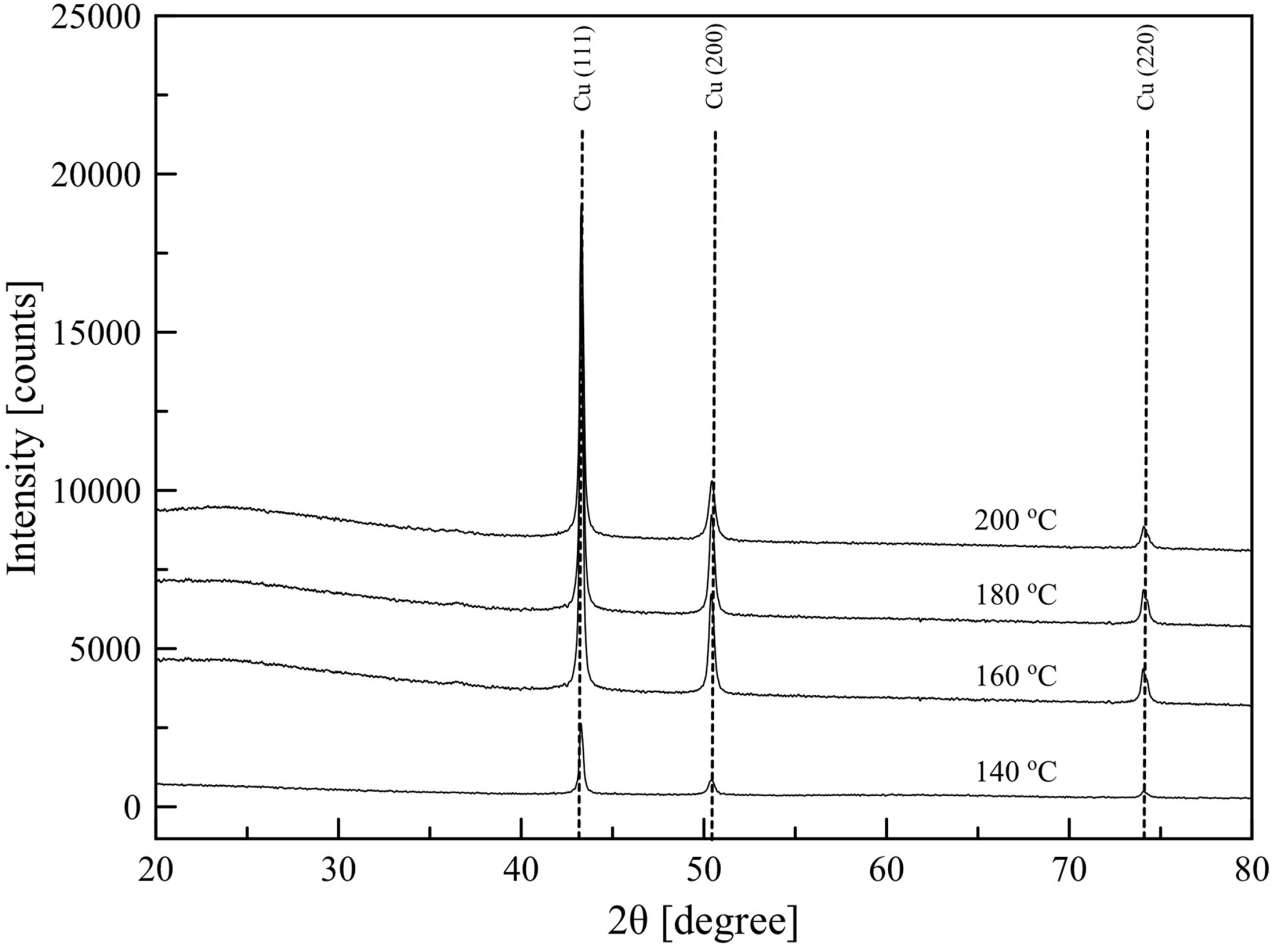
XRD patterns of the samples fabricated using 0.35 M Cu-ion with spraying rate 2 mL/min (spraying time 5 min) and annealing time 20 min at various temperatures.

The composition of the deposited copper patterns was confirmed using crystallographic analysis with XRD. Fig 3 shows the XRD patterns of the patterns fabricated using 0.35 M ink at 140°, 160°, 180° and 200° with spraying rate 2 mL/min and annealing at the same temperature for 25 min. The patterns were compared with the peaks of copper reported by Joint Committee on Power Diffraction Standards (JCPDS 03-1018). For all cases, peaks at 43.4°, 50.5°, and 74.1° were observed, indicating that the obtained patterns contained metallic copper. Also, the slight appearance of Cu_2_O at 36.4° was observed. This peak may be resulted from native oxide layers on the surface of the patterns. Generally, a copper film readily forms oxide layers on its surface due to its high reactivity with oxygen. Beside the peak at 36.4 °, no diffraction peaks resulted from other impurities were detected. Furthermore, the average crystallite sizes of the patterns were using the Scherrer’s formula. The patterns deposited at 140, 160, 180 and 200 °C had average crystallite sizes of 24, 26, 30, and 32 nm, respectively. The XRD diffraction peaks of the pattern fabricated at lower temperature indicated small size of crystalline nanoparticles. The morphology of the patterns was further analyzed using SEM images.

**Fig 4:**
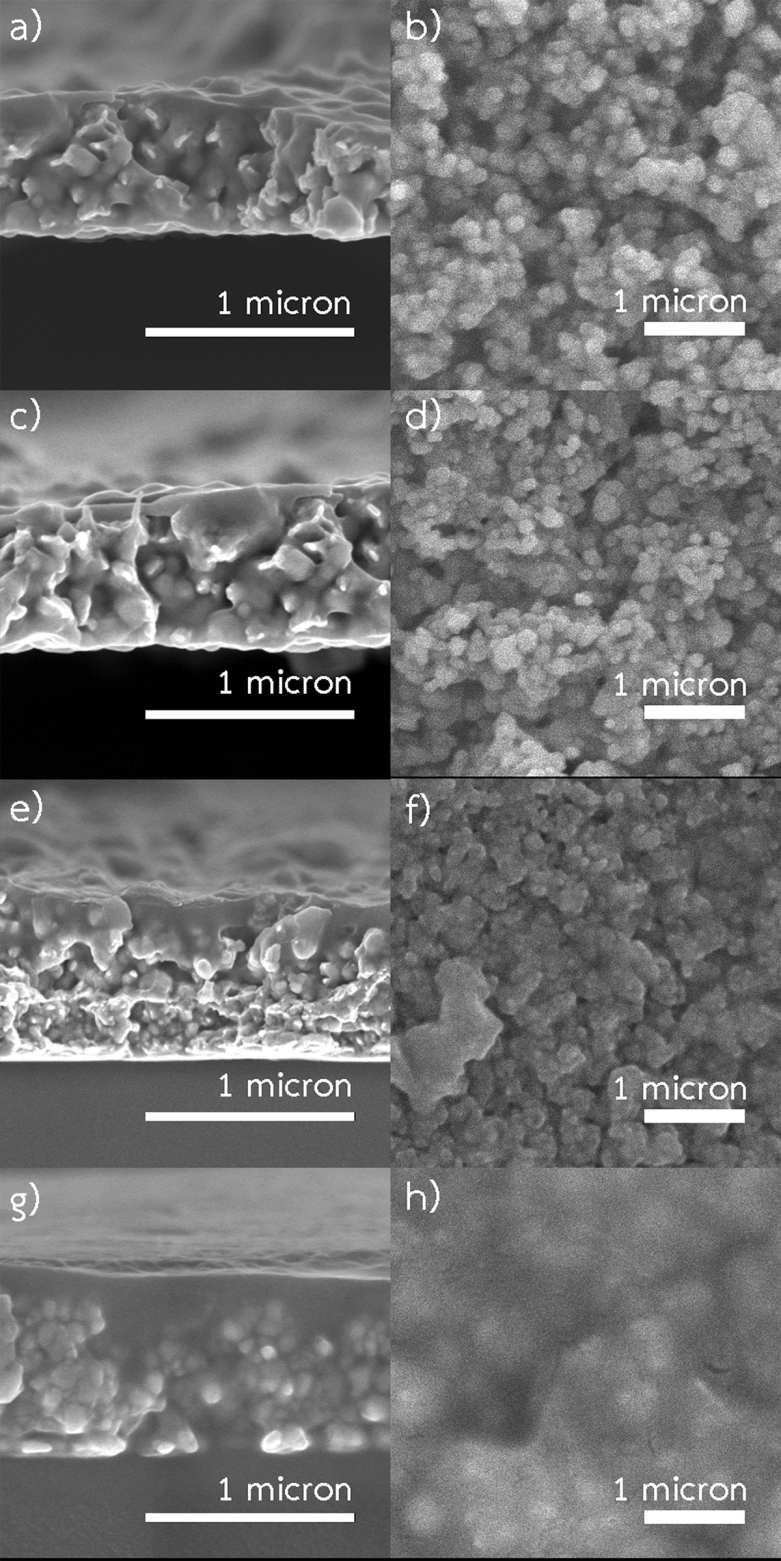
Cross-sectional and surface SEM images of the samples fabricated using 0.35 M Cu-ion with spraying rate 2 mL/min (spraying time 5 min) and annealing time 20 min. (a) cross-sectional image of the samples fabricated at 140 °C. (b) surface images of the samples fabricated at 140 °C. (c) cross-sectional image of the samples fabricated at 160 °C. (d) surface images of the samples fabricated at 160 °C. (e) cross-sectional image of the samples fabricated at 180 °C. (f) surface images of the samples fabricated at 180 °C. (g) cross-sectional image of the samples fabricated at 200 °C. (h) surface images of the samples fabricated at 200 °C.

Fig 4 shows SEM images of the samples fabricated at 140, 160, 180 and 200 °C. In all cases, the patterns composed of interconnected small copper particles with a few void spaces. Moreover, the grain boundaries were still apparently noticeable. The pattern fabricated at 200 °C was smoother and denser than the patterns fabricated at lower temperatures. Moreover, the pattern composed of two layers including a well-sintered layer on the surface, and the bottom layer with a lower sintering degree of copper particles. In contrast, a layer with high sintering degree was not observed in the patterns fabricated at lower temperatures.

**Fig 5:**
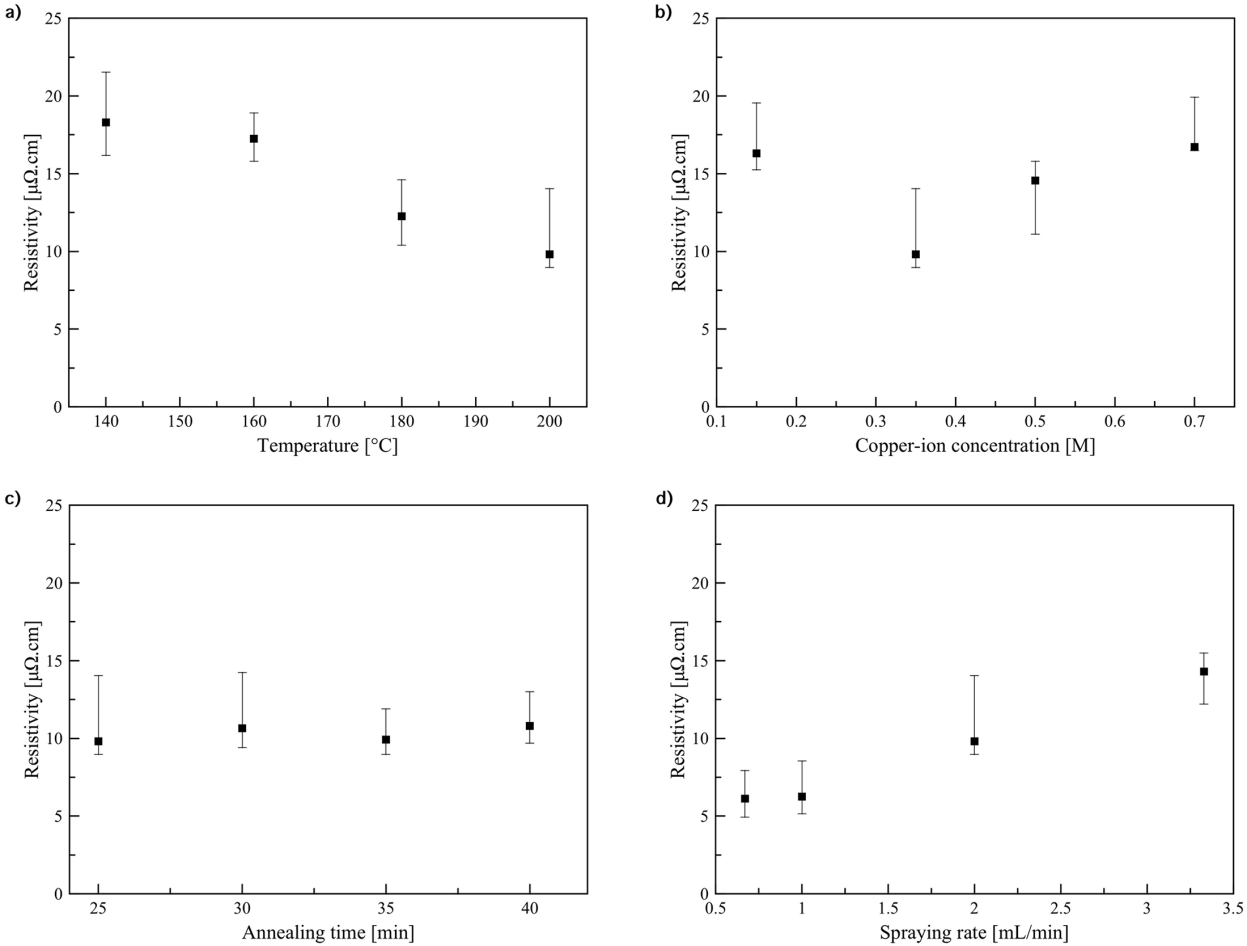
Variation of resistivity of the patterns. (a) the patterns fabricated using 0.35 M Cu-ion with spraying rate 2 mL/min (spraying time 5 min) and annealing time 20 min at various annealing temperatures. (b) the patterns fabricated using spraying rate 2 mL/min (spraying time 5 min) and annealing time 20 min at 200 °C and various Cu-ion concentrations. (c) the patterns fabricated using 0.35 M Cu-ion with spraying rate 2 mL/min (spraying time 5 min) at 200 °C and various annealing times. (d) the patterns fabricated using 0.35 M Cu-ion with annealing time 20 min at 200 °C and various spraying rates.

The resistivity of the pattern fabricated at 200 °C was significantly lower than those fabricated at lower temperatures. The variation of resistivity of the patterns is shown in Fig 5a. During spray-pyrolysis, tiny copper particles were generated inside small aerosol droplets and deposited on the substrate. Then, sintering between these particles occurred. Thus, a connected and conductive network was formed. On the one hand, at higher temperature smaller size particles are usually produced due to the formation of more nuclei caused by a more rapid reaction. Consequently, the pattern is tightly packed, however, the number of grain boundaries grows leading to higher resistivity. On the contrary, the higher temperature generally promotes the sintering between these particles, leading to decreasing of the resistivity. As observed from SEM images in Fig 4g and 4h, the spray-pyrolysis temperature of 200 °C yielded the pattern with higher sintering degree.

### Effects of concentration of copper-ion

**Fig 6:**
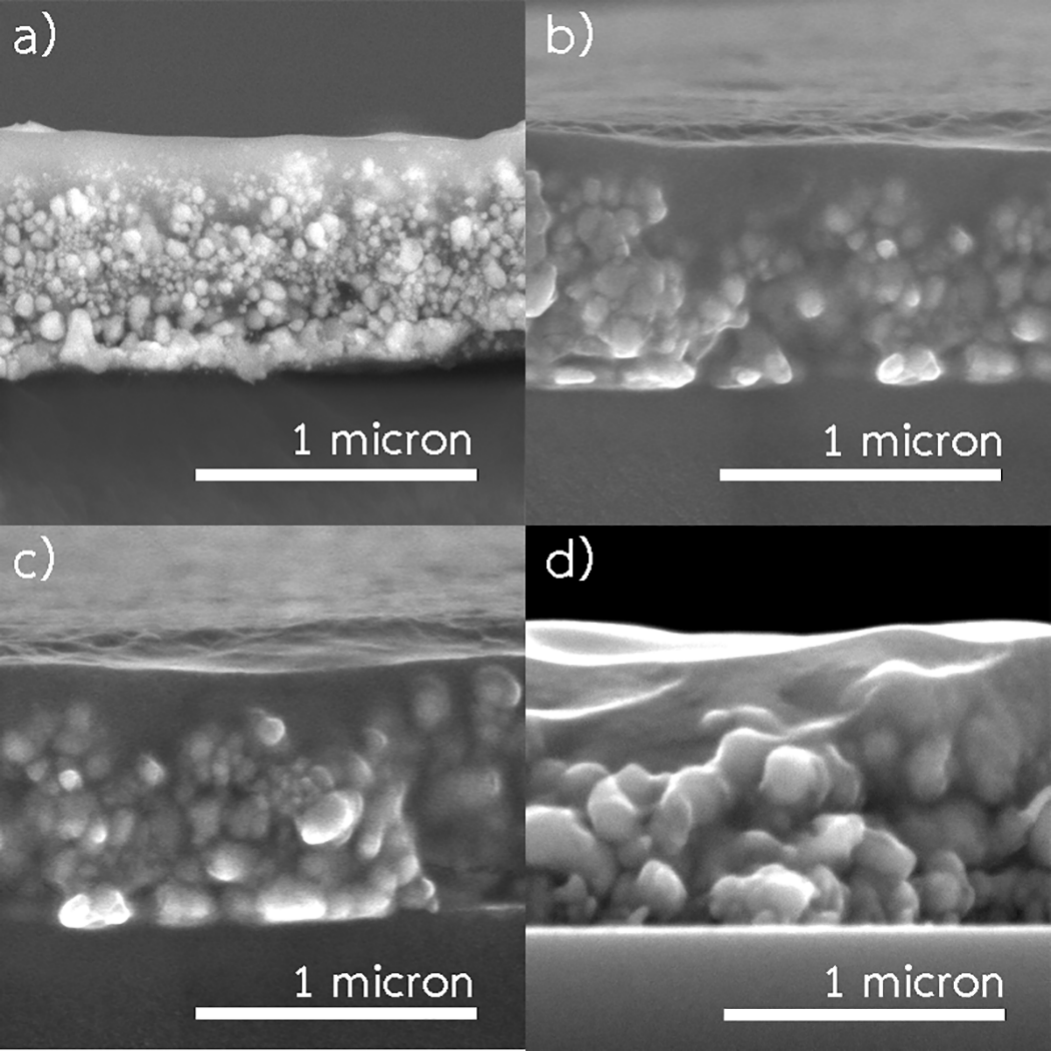
SEM images of the patterns fabricated using spraying rate 2 mL/min (spraying time 5 min) at 200 °C and annealing time 20 min. (a) 0.15 M Cu-ion concentration, (b) 0.35 M Cu-ion concentration, (c) 0.50 M Cu-ion concentration and (d) 0.70 M Cu-ion concentration.

The precursor solution is also a significant parameter. Concentration of copper-ion influences properties of the patterns. Here, the patterns were fabricated at 200 °C using 25 min of an annealing time to investigate the effects of copper-ion concentration. Fig 6 displays SEM images of the patterns fabricated using different concentrations of copper-ion. Apparently, the pattern, produced using high concentration, contained large copper particles. Though it had lower number of grain boundaries, the size of void spaces increased. In contrast, using lower concentration, relatively smaller copper particles were generated. The resulted pattern had higher number of grain boundaries but lower porosity. Therefore, a trade-off between these two contradicting phenomena is essential.

Fig 5b shows resistivity of the patterns fabricated using different copper-ion concentrations. The 0.35 M ink yielded the lowest resistivity pattern. That is, the optimal trade-off between the number of grain boundaries and porosity was obtained using 0.35 M copper-ion concentration. In each case the resulted pattern consisted of two layers with different degree of sintering. Further improvement of conductivity of the patterns requires optimizing annealing conditions to facilitate sintering between generated copper particles.

### Effects of annealing time

**Fig 7:**
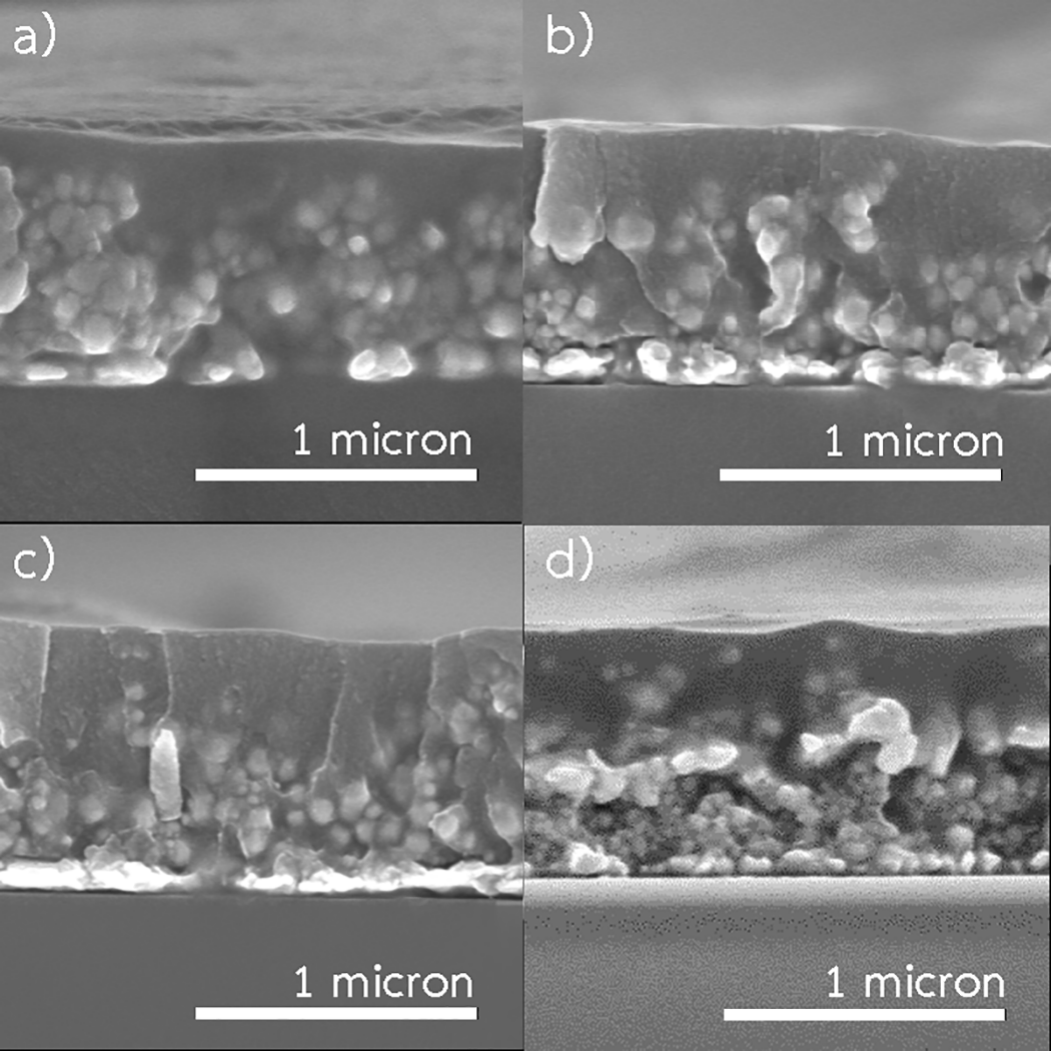
SEM images of the patterns fabricated using 0.35 M ink with spraying rate 2 mL/min (spraying time 5 min) at 200 °C. (a) 25 min annealing time. (b) 30 min annealing time. (c) 35 min annealing time. (d) 40 min annealing time.

The patterns were fabricated at 200 °C using 0.35 M ink at 200 °C with spraying rate 2 mL/min. Fig 7 presents SEM images of the patterns resulted from various durations of annealing. It was found that densification and crystallization of the patterns were almost completed only on the top layer. However, in the bottom layer, densification of the patterns resulted from necking between the particles was observed. Moreover, the variation of annealing time did not show any significant effects on sintering degree or the thickness of the top layer, which is a well-sintered layer, of the patterns. The results were in accordance with the resistivity as shown in Fig 4c. The resistivity did not change considerably with duration of annealing.

As proposed by Greer et. al [38], thermal sintering involves several atomic diffusion phenomena including surface diffusion, lattice diffusion, through lattice diffusion, and grain boundary diffusion, where the driving force is the reduction of surface energy. The surface diffusion, and lattice diffusion do not cause densification. However, densification is resulted from through lattice diffusion, and grain boundary diffusion, where the atoms are detached from the contact surface and move towards one another. Generally, in the initial stage of sintering, surface diffusion dominates the others because it has the easiest transport path and low activation energy. In addition, grain boundary diffusion usually prevails lattice diffusion because the activation energy for lattice diffusion is much greater than that for grain boundary diffusion. In other words, the atomic mobility within a grain boundary is much higher than that in the lattice.

In our system, as small copper particles were generated and deposited on the surface, sintering between these particles then occurred. The particles grew larger and formed necked contacts with each other. However, the particles did not have enough time and energy to complete sintering through densification and crystallization. After spraying, the patterns were thermally annealed inside the chamber. During this stage, heat transported from the surface of the patterns again induced sintering. However, recrystallization of growth grains inside the patterns is required to achieve a higher degree of sintering [39, 40]. Recrystallization also involves through lattice diffusion, and grain boundary diffusion. In addition, for large particles, the energy required for recrystallization is relatively high. Thus, even using annealing time of 40 min, significant effects on the degree of sintering could not be sought.

### Effects of spraying rate

**Fig 8:**
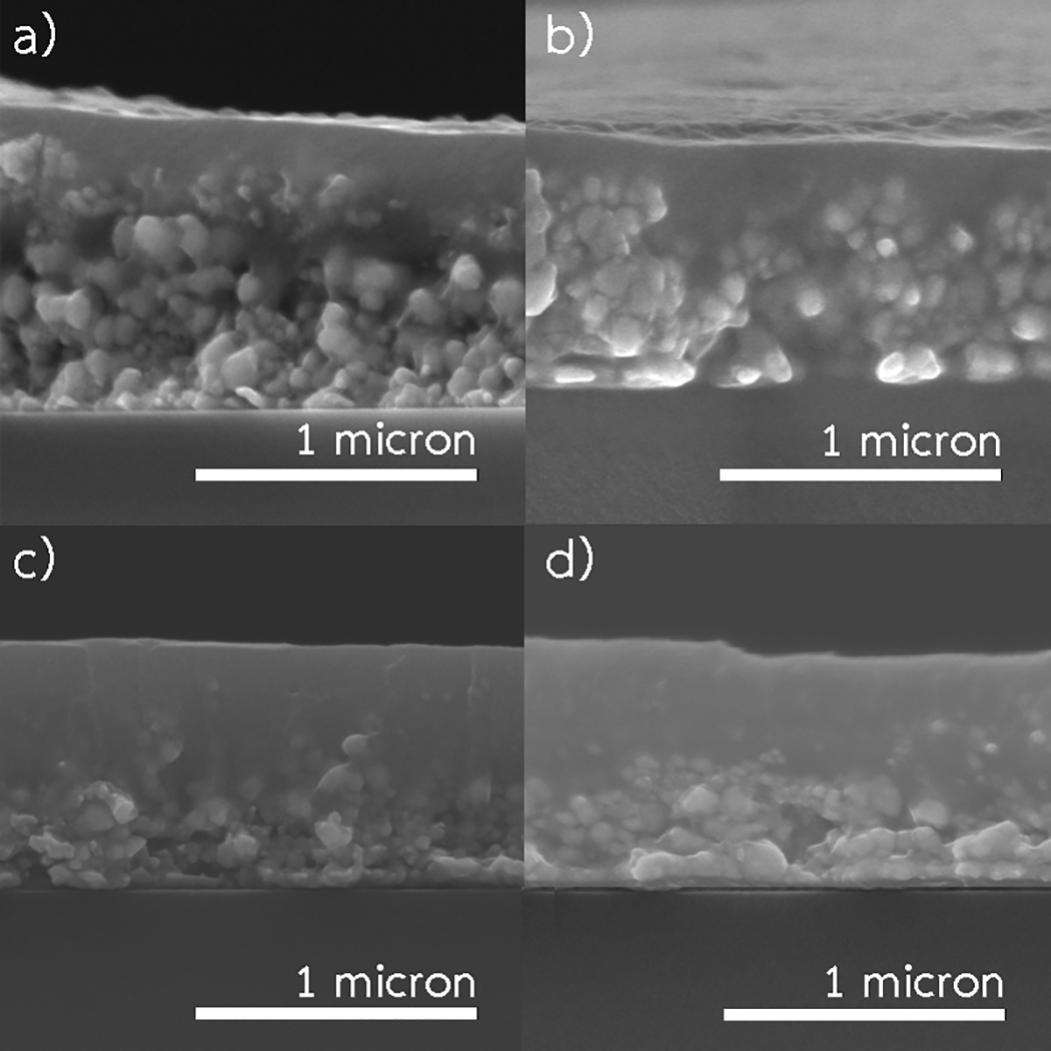
SEM images of the patterns fabricated using 0.35 M ink with 20 min annealing time at 200 °C. (a) spraying rate of 3.33 mL/min. (b) spraying rate of 2.00 mL/min, (c) spraying rate of 1 mL/min, (d) spraying rate of 0.67 mL/min.

The patterns were fabricated using 0.35 M ink at 200 °C with annealing time of 25 min. Four different spraying rates (0.67, 1, 2 and 3.33 mL/min) with corresponding spraying times (15, 10, 5 and 3 min) were investigated. In other words, the patterns fabricated using spraying rates of 0.67, 1, 2 and 3.33 mL/min were totally exposed to a temperature of 200 °C for 40, 35, 30 and 28 min, respectively. Fig 8 shows SEM images of the patterns resulted from various spraying rates. Using spraying rates of 3.33 and 2 mL/min, the patterns composed of two layers including a layer with high degree sintering on the surface, and the bottom layer with a lower degree of sintering. The thickness of the layer with high sintering degree was significantly increased by lowering the spraying rate, in other words increasing spraying time. Apparently, the lower spraying rate yielded a higher degree of sintering. Fig 4d displays the resistivity of the patterns fabricated using different spraying rates. The resistivity of the patterns dropped considerably with decreasing of spraying rate. However, spraying rate lower than 1 mL/min did not significantly reduce the resistivity. The lowest resistivity of the pattern was 6.12 *μ*Ω.cm which is about 3.6 times higher than the resistivity of bulk copper.

By using a low spraying rate, tiny copper particles, generated from decomposition of the ink, had a higher amount of energy and longer time to deposit and completely sinter into the pre-deposited layers. Although the pattern, fabricated using low spraying rate, was exposed to a temperature of 200 °C longer than those fabricated using high spraying rate, the longer exposure time is not the key contribution to achieving the low resistivity. As previously discussed, Fig 4c clearly indicated that the period (more than 25 min) of the exposure to a temperature of 200 °C did not enhance the conductivity. Therefore, the improved conductivity could result from the smaller particle formation with a low spraying rate. As shown in Fig 8c and 8d, by using low spraying rate, the well-sintered layer of the patterns was thicker than those fabricated using high spraying rate. Also, the patterns, fabricated using low spraying rate, exhibited lower resistivity. Thus, the spraying rate is an important parameter defining the degree of sintering as well as the conductivity.

## Conclusion

Fabrication of copper conductive patterns using low-temperature spray-pyrolysis of a copper ink, mainly composed of copper formate and diethanolamine, under N_2_ flow at the maximum temperature of 200 °C was demonstrated. The patterns composed of two layers including a well-sintered layer on the surface, and the bottom layer with a lower sintering degree of copper particles. Spraying rate is a critical parameter defining the degree of sintering. The pattern fabricated using 0.35 M ink with spraying rate of 0.67 mL/min and annealing at 200 ° for 25 min exhibited the lowest resistivity of 6.12 *μ*Ω.cm which is about 3.6 times higher than the resistivity of bulk copper. This technique has a great potential in fabricating low cost electronic devices. Also, the study suggested the way to fabricate thin films with high degree of sintering using spray-pyrolysis.

## Supporting information

S1 File Thermogravimetric and differential thermal analysis (TG/DTA) data of the inks with various concentrations of Cu-ion. (XLSX)
